# Preservation of Hydrogen Peroxide

**Published:** 1898-02

**Authors:** 


### Preservation of Hydrogen Peroxide.

Prof. Scoville in writing of hydrogen peroxide quotes Zinno as stating that the addition of 0.1 percent of crystallized naphthalene to the solution materially retards decomposition, which claim he says has been confirmed by Shoen. The time-rate of decomposition is not stated. Prof. Scoville says that if filtration is necessory, pyroxylin is stated to be the filtration medium, since asbestos decomposes the peroxide and other media react with it.—*N. E. Druggist.*

